# Restraint use in home care: a qualitative study from a nursing perspective

**DOI:** 10.1186/1471-2318-14-17

**Published:** 2014-02-05

**Authors:** Kristien Scheepmans, Bernadette Dierckx de Casterlé, Louis Paquay, Hendrik Van Gansbeke, Steven Boonen, Koen Milisen

**Affiliations:** 1 Nursing Department, Wit-Gele Kruis van Vlaanderen, Brussels, Belgium; 2 Department of Public Health and Primary Care, Centre for Health Services and Nursing Research, KU Leuven, Kapucijnenvoer 35, 4th Floor, 3000 Leuven, Belgium; 3 Division of Geriatric Medicine, Leuven University Hospital, Herestraat 49, 3000 Leuven, Belgium; 4 Leuven University Center for Metabolic Bone Diseases, KU Leuven, Leuven, Belgium

**Keywords:** Home care, Physical restraints, Nursing

## Abstract

**Background:**

Despite the growing demand for home care and preliminary evidence suggesting that the use of restraint is common practice in home care, research about restraint use in this setting is scarce.

**Methods:**

To gain insight into the use of restraints in home care from the perspective of nurses, we conducted a qualitative explorative study. We conducted semi-structured face-to-face interviews of 14 nurses from Wit-Gele Kruis, a home-care organization in Flanders, Belgium. Interview transcripts were analyzed using the Qualitative Analysis Guide of Leuven.

**Results:**

Our findings revealed a lack of clarity among nurses about the concept of restraint in home care. Nurses reported that cognitively impaired older persons, who sometimes lived alone, were restrained or locked up without continuous follow-up. The interviews indicated that the patient’s family played a dominant role in the decision to use restraints. Reasons for using restraints included “providing relief to the family” and “keeping the patient at home as long as possible to avoid admission to a nursing home.” The nurses stated that general practitioners had no clear role in deciding whether to use restraints.

**Conclusions:**

These findings suggest that the issue of restraint use in home care is even more complex than in long-term residential care settings and acute hospital settings. They raise questions about the ethical and legal responsibilities of home-care providers, nurses, and general practitioners. There is an urgent need for further research to carefully document the use of restraints in home care and to better understand it so that appropriate guidance can be provided to healthcare workers.

## Background

Despite increasing evidence of negative consequences [1-4], the use of physical restraints is still common practice in many countries. The prevalence ranges between 4% and 85% in nursing homes [4] and between 8% and 68% in hospitals [5]. This wide range partly reflects varying definitions for what constitutes restraint, different populations studied, and different countries with differences in legislation and practice.

Due to shifting demographic, economic, and technological trends and the desire of patients to live at home as long as possible, home care is growing in demand. With these trends, an increasing number of frail older persons are living at home despite major cognitive disturbances and functional disabilities [6,7], conditions known to be associated with an increased use of restraints [4,5]. As a consequence, healthcare workers are increasingly confronted with restraint use, even in home care. Research on this topic in home care is scarce. One study conducted in the Netherlands suggested that restraint use in home care is common practice [8].

The use of restraints has a large impact on patients (e.g., physical and psychological consequences); family (e.g., anger, worry); and healthcare workers (e.g., mixed emotions such as anger, reassurance) [1,4,5,9]. Furthermore, the decision process to use restraints lies along a complex trajectory that depends on patient characteristics and on the attitude of nurses (e.g., nurses’ perception of patient behavior, their willingness to take risks, or their own comfort). It also depends on context-related factors such as family involvement, which can have a positive or negative impact on nurses’ decision making; insufficient time to discuss restraint use with other staff members like physicians; lack of staff; and the requirement of balancing safety, and ethical and legal aspects [4,10,11].

Current understanding about restraint use derives mostly from acute and long-term residential settings. Knowledge about restraint use obtained from residential settings does not easily generalize to the home-care setting, because of the uniqueness of the home-care setting. Moreover, it is unclear how the little research that has been done in home-care settings relates to that done in long-term residential care settings. In the home-care setting, healthcare professionals work in the patient’s personal living environment rather than in a healthcare facility, where they have more control over decisions. Moreover, they see their patients for short visits; thus, they have no opportunity to continuously supervise restrained persons. Also, home-care nurses typically work alone, often leaving them in an unsupported professional position when confronted with decisions about restraints. Patients’ relatives also play a crucial role and may even take the lead in the decision-making process [12,13]. These factors emphasize the need for research on restraint use in home-care settings.

Because of their pivotal role in home care and their intensive interactions with family and other healthcare workers, home-care nurses are in an excellent position to provide relevant information about the use of restraints in home care. The aim of this study was to gain initial insights into the use of restraints in home care in Flanders, Belgium, from the perspective of home nurses. The overarching research question was, “What are nurses’ experiences about restraint use in Flemish home care?”

## Methods

### Design

A qualitative explorative study was performed to gain more insight into the experiences nurses have with restraint use in the home-care setting [14,15]. Physical restraint is defined as using “any device, material or equipment attached to or near a person’s body and which cannot be controlled or easily removed by the person and which deliberately prevents or is deliberately intended to prevent a person’s free body movement to a position of choice and/or a person’s normal access to their body” [16]. We extended this definition to include other forms of restraint; e.g., chemical and environmental restraints and any other action applied by someone that restricts another person’s freedom in some way.

### Setting

The study was conducted in the Wit-Gele Kruis, an umbrella organization that provides home nursing in Flanders, Belgium. In Belgium, professional home nursing is provided by a private organization, an agency, or by self-employed nurses. Organizations, such as the Wit-Gele Kruis, have a similar organizational structure to a hospital: nursing director, management head, and nurses. All nurses working at Wit-Gele Kruis are employees and provide care for patients living at home. Professional home nursing is part of the social security system and is financed by the National Institute for Health and Disability Insurance (NIHDI). This institution reimburses patients who are insured, which is mandatory in Belgium. Furthermore, the NIHDI reimburses for a limited set of nursing activities listed in the nomenclature for home nursing. This list of home nursing activities has codes that correspond to an honorarium or reimbursement fee [17]. Most nursing care activities must be prescribed by a physician to be reimbursable. However, no prescription is required for the use of restraints in this nomenclature, which refers to a nursing activity as “measures to prevent injury” and “includes restraint devices, insulation, security, and surveillance.” In short, this means that nurses can perform these kinds of actions under certain conditions and that they bear responsibility for its implementation.

Wit-Gele Kruis consists of five autonomous home nursing agencies, each of which is located in one of the five Flemish provinces and is spread over 100 divisions. In 2012, 153,199 patients received at-home nursing care from Wit-Gele Kruis. The mean age of these patients was 72.9 years, with 80.3% being older than 60 years.

### Participants

The head nurses of nine randomly selected divisions were contacted and informed about the aim of our study. They were asked to select home-care nurses who met the following criteria: (i) delivered direct patient care at home, (ii) had experience with the use of restraints at home, and (iii) were willing to talk about their experiences. The researcher contacted potential candidates to confirm their voluntary participation and to set a date for in-depth-interviews. All participants gave written informed consent.

The purposive sample consisted of 14 nurses (13 females) who had an average age of 39 years (range: 23–57 years) and an average of 11.4 years (range: 11 months - 24 years) of professional experience as a home-care nurse. Eight of them worked full time.

To become a nurse in Belgium, one can chose from two types of training or educational programs: baccalaureate-level or associate-level nursing programs. Nurses with baccalaureate degrees graduated from a nursing program at a college for higher education. Nurses with an associate degree received polytechnical nurse training in their fourth year of secondary school. Of the 14 participants, 6 were nurses with a baccalaureate degree and 8 were nurses with an associate degree.

### Procedure

Data were collected from April to June 2009, using semi-structured in-depth interviews. Each interview took approximately 1-1½ hours and was conducted at the division where the participant was employed. All interviews were digitally recorded. The first author (K.S.) conducted all interviews and had no professional relationship with the participants.

The interview guide consisted of open-ended questions and was refined throughout the research project (Table 1). We started the interview by asking the respondents to describe the concept of “restraints” in their own words. Next, we asked the nurses to provide a specific example of a situation in which they had used restraints in home care. The questions listed in Table 1 helped us to gather more information about their experiences.

**Table 1:** Interview guide

	
– Please give an example of a situation in which you were faced with the use of restraints?	– What difficulties did you experience by using restraints?
– What types of restraints did you use in this situation?	– Can you describe how you dealt with this situation and why?
– What was the reason for using these restraints?	– Who supported you in this situation?
– Were these restraints used in an acute or chronic way?*	– Can you explain the role of the general practitioner in using restraints?*
– Can you explain how the decision about restraint use was made (e.g., during team meetings)?*	– What were available alternatives in this situation?*
– How did you experience the use of restraints?	– In your opinion, what would be the best care for this patient?
– Can you describe your emotions when using restraints?	– As a nurse, how did you experience your responsibility in this situation?

*Additional questions after the initial interviews.

The interview guide was adapted and refined according to insights made from the first interviews. The research team also added some questions to gather more information about the general practitioner’s role, nurses’ knowledge of available alternatives, and the organization of team meetings. The goal of this was to better understand the decision-making process, and whether restraints were used acutely or chronically. The order of the questions was adapted according to the answers of each nurse during his/her interview. After discussing the first example of restraint use, we asked the nurses to provide another example of restraint use and to explain how this differed from the previous situation. We also asked about other kinds of restraints used in the home-care setting. Finally, we asked them to describe an ethically irresponsible situation they experienced and how they dealt with that situation.

### Ethical approval

The Medical Ethics Committee of the Leuven University Hospitals approved the study.

### Data analysis

The data were analyzed using the method described in the Qualitative Analysis Guide of Leuven (Quagol) [18]. Data collection and thematic analyses occurred in parallel, with continuous interaction between the two. Much time was devoted to analyzing and understanding the data and thoroughly preparing the coding process.

Each tape-recorded interview was transcribed verbatim and read several times in order to obtain a general picture of restraint use in home care and to make sense of the material provided by the nurses. Significant statements were extracted and codes/concepts were formulated that conveyed the essential meaning of the nurses’ experiences. Statement fragments with similar codes were ordered and organized into categories per interview. These categories were then compared with what was said in the original interviews. After analyzing each interview separately, the research team determined common categories produced across the different interviews. This resulted in a master list of concepts/codes, which served as the data that was entered into the qualitative software program Nvivo 7.0.

All interviews were analyzed by the interviewer. In addition, the first interviews were read and analyzed by all members of the research team and discussed in a group. The remaining interviews were divided among three members of the research team, so that each interview was read, significant statements were indicated and coded by two members and the interviewer. All findings were discussed by the team and always verified with the interviews.

The research team consisted of four members. The members had a mixture of expertise in the field of home care, restraints, qualitative research, and ethics. Analysis started immediately after the first interview and continued until saturation was reached. Several strategies (researcher triangulation, context triangulation, audit-trail, peer-debriefing) were used to optimize the methodological quality of the study [19].

## Results

The present study confirmed that restraints are used in home care, but at the same time, revealed a lack of clarity about this concept among nurse participants. Furthermore, the study showed that the use of restraints was associated with specific features unique to this type of setting, including types of restraints used, patient characteristics, reasons for restraining, and persons involved in the decision-making process.

### Restraints in home care: an ambiguous concept

The participating nurses had difficulty in defining the use of restraints. The interviews revealed a variety of interpretations of this concept related to particular personal and professional experiences of the nurses. Some nurses even considered home modifications, like moving the bed downstairs, to be a form of restraint. Other nurses had a very restricted interpretation of restraint use, more in line with the notion of abuse or neglect of older individuals. Between these two extremes, activities like turning off the gas for cooking, the use of sheets, bedrails, a geriatric chair, etc., were interpreted by some nurses to be a use of restraints.

There was also confusion between the concepts of “restraints” and “safety measures.” Many measures like the use of bedrails or a geriatric chair - even without the patient’s approval - were considered to be safety measures, not restraints.

During the interviews, we noted that the participating home-care nurses became increasingly aware of the meaning of “restraints” and its use in daily clinical practice.

“The questions stimulate you to think; normally you don’t realize it. When the question is asked, you start thinking and then you see the concept a lot larger. I thought that I could not tell a lot because I had not been confronted with restraints, but suddenly I realized I could give many examples.” (Interview 2)

### Characteristics of restraint use in home care

#### Types of restraints

Commonly used restraints, like geriatric chairs, belts, bedrails, and other types of restraints were used. Nurses reported limiting patients’ freedom of movement by restricting access to stairs, by reorganizing areas in the house, by putting away medication, by turning off the gas, and by locking the front door. They also reported systematically locking the patient in a separate room. These were typical examples of the use of restraints in home care. According to the nurses, medication to control behavior (chemical restraint) was often administered by the family.

#### Patient characteristics

In home care, restraints were most frequently used for older persons experiencing cognitive decline (e.g., patients with dementia). Often these patients lived alone and had no family nearby or other forms of supervision.

“*I have a patient who is demented, according to the family. In my opinion she is slightly demented. After each care I must lock her up, put the key away and leave. The patient sits by the window, watches me, and rattles the door. This is really difficult.” (Interview 8)*

#### Reasons for using restraints

In addition to ensuring the safety of a patient at home, “keeping the patient at home as long as possible” was a common reason nurses gave for using restraints in this setting. Often for financial reasons, restraints were used as a tool to avoid admitting the patient to a nursing home.

“Without restraints, it is not possible to keep her at home, and she will have to go to a nursing home. Because of the distance and her husband’s bad health, this would make it impossible for him to visit her.” (Interview 1)

“Because people have no other choice. I think that when this patient goes to a nursing home, the same will happen. Besides, they will give the patient more medication to calm him than when he lived at home.” (Interview 5)

“We have a key to the patient’s home. After the care, we lay her in bed with bedrails and lock the front door. This is for her safety. Otherwise, she would need to go to a nursing home, which scared her a lot.” (Interview 11)

Another specific reason for using restraints at home was to relieve the informal caregiver. Nurses emphasized that caring for a cognitively impaired older person is exhausting. Restraints allowed patients’ family to do other things like shopping and provided some respite, since with restraints they wouldn’t have to look constantly after their relatives.

“I often see that restraints are used to protect their informal caregiver/neighbor, to limit their stress. They apply restraints not for the safety of the patient but to relieve relatives and themselves.” (Interview 2)

#### Persons involved in the decision-making process

The family appeared to play an important role in the decision-making process on whether to use restraint, either facilitating or complicating the process. In most cases, family members and nurses worked together to find the best solutions. Sometimes family played a dominant role and made their own decision, thereby putting some nurses in a difficult situation, especially when the demands of the family were in conflict with the patient’s well-being. Because nurses were considered to be “visitors” in the home of the patient and their family, they often felt obliged to accept the dominant role of the patient’s family.

“Often it is the family who takes the initiative to [use] restraints, when they can no longer deal with the situation. For example, I knew a family who used a sheet as a belt to protect the patient from falling.” (Interview 3)

“When the family asks for something, you cannot refuse it. When the children ask to restrain a patient, we typically comply with their request, because this is home care and we depend on the family.” (Interview 8)

An unexpected finding that emerged from the interviews was that nurses did not mention the patient’s general practitioner, unless they were asked to do so. When specifically asked, the nurses implied that the general practitioner had no role in the decision to use restraints, except when asked to prescribe medication to control the patient’s behavior. Nurses reported that they would prefer the general practitioner to play a more active role because of his/her prominent and respected position.

“I see the general practitioner as someone having more influence on the family.” (Interview 6)

## Discussion

To the best of our knowledge, this is the first study to show from the perspective of home-care nurses that restraint use in home care is definitely an important issue. During the interviews, we noticed that the nurses became increasingly aware of the full meaning of the “restraints” concept and the consequences of restraint use. Discussing restraint use helped us to better understand the concept and its implementation in the home-care setting. The absence of a clear policy on restraint use within our organization, as confirmed by the participating nurses, typically contributed to ignorance or confusion at the beginning of the interview.

In line with our findings, de Veer et al. [8] also emphasized the importance of a documented policy in home-care organizations that offer educational programs for nurses and other healthcare workers. Such documentation provides guidance for everyone involved on how to ensure safety and use appropriate surveillance. Furthermore, it helps direct solutions when family members have different opinions.

Our findings also underscore the need to initiate careful study on the prevalence of restraint use in home care. Taking into consideration the expressed ambiguity around the concept, a clear operationalization of the concept of restraint use in home care will be required in future studies. The results of the current study can serve as an important basis for developing a new questionnaire to study the prevalence of restraint use, one adapted to the unique features of the home-care setting.

Results of our study also prompt ethical and legal questions because of the documented absence of continuous follow-up, the dominant role of family, and the specific reasons for using restraints in home care. Our interviewees reported that patients were restrained or locked up and left alone, which is at variance with best practice guidelines and underscores the need for supervision when patients are restrained [20]. Medication to control a patient’s behavior (i.e., neuroleptic drugs, benzodiazepines, etc.) was often given by the family with no or minimal professional follow-up. This disturbing finding raises questions about the ethical and legal responsibilities of home-care providers, nurses, and general practitioners. The absence of a clear role of the general practitioner in restraint use, as reported by the nurses, should be further explored. In Belgium, like in most European countries, general practitioners are supposed to play a central role in primary home care.

The dominant role of the family in home care may pose major challenges to care providers. For example, at times, relatives insist on the use of restraints or certain types of restraints, which, according to the nurses, do not contribute to “good” care. Providing care that does not promote the overall feeling that the patient is a human being in all dimensions (physical, relational, social, psychological, moral, and spiritual) is considered by nurses to be a morally distressing situation [21]. Employers of agencies should provide a clear policy or guidance to staff on the use of restraints, focusing on a multi-disciplinary approach to individual care planning that includes risk assessment procedures and appropriate education, among other training and guidance. This will help nurses and other staff to make appropriate decisions about the use of restraints [22].

One of the reasons reported for using restraints was to keep the patient at home as long as possible and to avoid admission to a nursing home. Respite for informal caregivers was another specific reason given for restraining a patient at home. Regardless of the reason, it is important that healthcare workers discuss the patient’s values with him or her. It is a challenge to choose and implement the best option that helps the patient feel like a human being.

The decision to use restraints should not be the sole responsibility of the family, but should be discussed by the whole team, including the patient and his or her family. In addition to focusing on the person being or not being restrained, it is essential to support the family in this decision-making process. Our interviews revealed that nurses are confronted with extremely difficult situations in which their opinions differ with those of the family. For example, they might question the motives of the family to use restraints, or they might disagree with the context in which restraints should be used, which types of restraints and materials are used, or how they are applied. Managers of home-care organizations need to be aware that nurses have to make difficult choices between organizational values, family demands, and what they personally consider to be morally right. Not being able to act according to personal ethical values ultimately causes nurses to experience moral distress [23]. These situations raise questions about the role, position, and responsibility of home-care organizations. A clear organizational policy serving as a firm basis for decision making is necessary.

This study presents the first qualitative research about restraint use from a home-care nursing perspective. The strengths of this study are the data analysis methods, which were characterized by a strong team approach (intensive analyses carried out by the entire research team), a case-oriented approach, and a forward-backward dynamics using a constant comparative method [19]. Sample heterogeneity (in terms of age and experiences of the nurses, who come from different divisions) contributed to saturation, except for data regarding the role of general practitioners.

This study also has limitations. The first limitation concerns the sampling strategy. We asked the head nurses of nine randomly selected districts to select home-care nurses who met the recruitment criteria. Voluntary participation of the home-care nurses may be questionable, because of perceived differences in professional power held by the in-home nurses and the head nurses. Nevertheless, we asked the nurses twice (during the first call and before starting the interview) whether their participation was voluntary, and emphasized that the interview and data analysis would remain anonymous and would not influence their professional activities. We also emphasized that the researcher had no hierarchical relationship with the management of the organization.

Another limitation of the study is the lack of depth in data collection, which resulted in data saturation after only 14 interviews. Initially, we planned to interview about 20 home-care nurses, depending on the point of saturation. Unfamiliarity with the concept of “restraint” (describing the concept took up a lot of time) and difficulty in discussing such a complex, ethically laden subject could have contributed to the lack of depth in the data.

A third limitation is that our analysis resulted in the identification of several major categories, rather than themes that would normally be expected on the basis of the current analysis process. Although the data did not allow a more in-depth analysis, nevertheless it revealed important information about the use of restraints in home care, in accordance with the purpose of the study.

This study focused only on the experiences of home-care nurses with regard to the use of restraints. However, the nurses’ perspectives on restraint use should be supplemented with the viewpoints and experiences of others involved. Further research on restraint use from the perspective of the patients’ family and physicians is needed to better understand the prominent role of the family and the expected role of general practitioners. Also, there is lack of information about the experiences of home-care patients themselves. This information is needed in order to develop an evidence-based practice guideline for proper management of restraint use in home care.

## Conclusions

This study provides insights into the use of restraints in home care from nurses’ perspective. Our results indicate that restraint use is an important issue and is frequently used in home care. It is possibly even more complex than in long-term residential care settings and acute hospital settings. There is an urgent need for further research to carefully document and understand the use of restraints in home care and the experiences of all persons and organizations involved.

## Competing interests

The authors declare that they have no competing interests.

## Authors’ contributions

KS was responsible for the study concept and design, acquisition of data, analysis and interpretation of data, and drafting the manuscript. BD, LP, and KM contributed to the study concept and design, the acquisition of data, the analysis and interpretation of data, and the drafting of the manuscript. HVG contributed to the study concept and design, and the drafting of the manuscript. SB participated in drafting the manuscript. All authors revised the manuscript. Supervision was done by KM. All authors read and approved the final manuscript.

## Pre-publication history

The pre-publication history for this paper can be accessed here:

http://www.biomedcentral.com/1471-2318/14/17/prepub
